# Brightfield vs Fluorescent Staining Dataset–A Test Bed Image Set for Machine Learning based Virtual Staining

**DOI:** 10.1038/s41597-023-02065-7

**Published:** 2023-03-22

**Authors:** Elena Y. Trizna, Aleksandr M. Sinitca, Asya I. Lyanova, Diana R. Baidamshina, Pavel V. Zelenikhin, Dmitrii I. Kaplun, Airat R. Kayumov, Mikhail I. Bogachev

**Affiliations:** 1 Institute for Fundamental Medicine and Biology, Kazan Federal University, Kazan, 420008 Russia; 2 Centre for Digital Telecommunication Technologies, St. Petersburg Electrotechnical University “LETI”, St. Petersburg, 197022 Russia

**Keywords:** Wide-field fluorescence microscopy, Fluorescence imaging

## Abstract

Differential fluorescent staining is an effective tool widely adopted for the visualization, segmentation and quantification of cells and cellular substructures as a part of standard microscopic imaging protocols. Incompatibility of staining agents with viable cells represents major and often inevitable limitations to its applicability in live experiments, requiring extraction of samples at different stages of experiment increasing laboratory costs. Accordingly, development of computerized image analysis methodology capable of segmentation and quantification of cells and cellular substructures from plain monochromatic images obtained by light microscopy without help of any physical markup techniques is of considerable interest. The enclosed set contains human colon adenocarcinoma Caco-2 cells microscopic images obtained under various imaging conditions with different viable vs non-viable cells fractions. Each field of view is provided in a three-fold representation, including phase-contrast microscopy and two differential fluorescent microscopy images with specific markup of viable and non-viable cells, respectively, produced using two different staining schemes, representing a prominent test bed for the validation of image analysis methods.

## Background & Summary

Rapid advancement of biomedical imaging technologies requires the development of efficient computerized image analysis methodology capable of segmentation and quantification of cells and cellular substructures from microscopic images without using any specific physical markup techniques. In turn, learning and validation of newly developed methods and algorithms ranging from computer vision to machine learning based solution requires adequate image sets with available “ground truth” segmentation. While multiple image sets are available for this purpose, a majority of them provide “ground truth” segmentation based on the manual expert assessments. However, for the development and validation of computerized alternatives to physical markup techniques such as differential fluorescent staining, an image set where the same fields of view are provided in both fluorescent channels representing the results of physical markup and monochromatic channels obtained by phase-contrast microscopy are the most suitable. The above problem could be also treated as the development of computerized technique to generate a surrogate image channel that could to a certain approximation replace the physical markup, without alteration of the rest of the experimental protocol.

Among available annotated biomedical image sets for testing and validation of computerized methods and algorithms, the Broad Bioimage Benchmark Collection (BBBC) represents a prominent example. Containing over 500 separate image sets with both prokaryotic and eukaryotic cells, including blast, embryonic, stem, synthetic cells, as well as macrophages, obtained by bright-field, phase contrast or fluorescent microscopy, the data represent a broad collection of various microscopic images^1^. Among other examples, various microscopic and reconstructed images of multiple cell lines of different origins and morphology acquired under various experimental setup and treatments including experiments conducted with time-lapse acquisition using quantitative phase-contrast microscopy (QPI) are represented in^2^. LIVECell^3^ is another example of manually annotated and expert-validated dataset of phase-contrast images, consisting of more than 1.6 million cells from a diverse variety of cell morphologies and cultures.

Multiple image sets based on various samples from different cancer research studies are available, such as, for example, 58 H&E-stained histopathology images of breast cancer cells used to train algorithms for cell segmentation and subsequent classification of benign and malignant cells^4^. Another relevant example is the C-NMC 2019 dataset containing morphological images associated with blood disorders and widespread types of childhood cancers was prepared at Laboratory Oncology, AIIMS, New Delhi and consists of 15,135 images from 118 patients^5^.

Despite a large number of available datasets, they vary considerably in the form of annotation provided, ranging from manual segmentation and/or cell type selection to cell counts only. In particular, the complete blood count (CBC) dataset^6^ contains 360 blood smear images along with their annotation files. Another example with 4600 images and 26 000 segmented cells collected in EVICAN-Expert visual cell annotation^7^, comprising partially annotated grayscale images of 30 different cell lines from multiple microscopes, contrast mechanisms and magnifications, with each image annotated by a certified pathologist to provide a knowledge base.

There are numerous examples of bright-field, phase-contrast and fluorescent microscopic images, including similar types of cells, but they are rarely provided in the same field of view, which does not allow to either train or validate the algorithms for the bright-field and/or phase-contrast image analysis using fluorescent microscopy data as a reference. Publication of this image set aims at providing a relevant test bed to allow investigators to learn and validate the best alternatives to the widely adopted physical markup techniques based on the analysis of light microscopic images that, in marked contrast to staining techniques, are compatible with viable cells and thus could be applied to live experiments.

## Methods

**Fig. 1:**
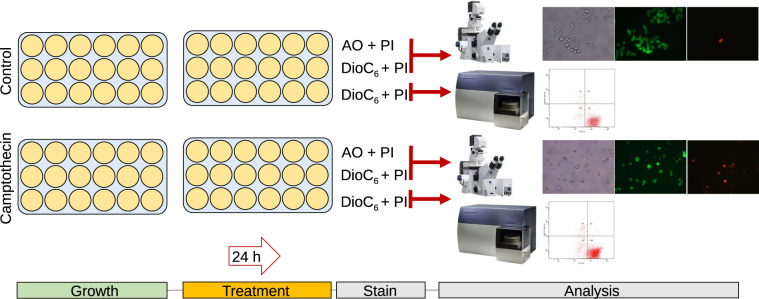
Overview of experimental design and study workflow. The Caco-2 cells were grown in 96-well plate until 70% monolayer. Then broth was changed, in half of wells the camptothecin (a topoisomerase inhibitor) was added and cultivation was followed for 24 h. Then cells were stained with either Acridine Orange (3 *μg/ml*) or DioC6 (0.02 *μg/ml*) and propidium iodide (3 *μg/ml*) and series of 3 wells were analyzed with either microscopy on Carl Zeiss Observer 1.0 microscope with 40× magnification or with flow cytometry using BD FACSCanto II flow cytometer.

The experimental design is summarized in Fig. 1. The human colon adenocarcinoma Caco-2 (ATCC HTB 37; ECACC 86010202) were obtained from Russian cell culture collection (Institute of Cytology, RAS, Saint-Petersburg, Russia). Cells were grown in 96-well plate in DMEM broth supplemented with 10% FBS and 2 mM L-glutamine. The penicillin (100 *μg/ml*) and streptomycin (100 *μg/ml*) were added to prevent bacterial contamination. The cells were seeded at the density of 3000 cells per well and cultured at 37 °C and 5% *CO*_2_ with broth change each two days until 70% monolayer. Then broth was changed, in half of wells the camptothecin (Sigma-Aldrich) was added in final concentration of 6 µM and cultivation was followed for 24 h. Then cells were stained with either Acridine Orange (3 *μg/ml*) or DioC6 (0.02 *μg/ml*) and propidium iodide (3 *μg/ml*) and series of 3 wells were analyzed with microscopy on Carl Zeiss Observer 1.0 microscope with 40× magnification. Low quality images due to technical issues such as overexposed background, pronounced distortion, sample out of focus and similar, have been excluded from further analysis, resulting in variable number of valid fields of view (see Table 1).

**Table 1:** Types and count of microscopy view fields in the dataset.

Series Name	Treatment	Samples count (view fields)	Staining	Dead cells	Dead cells fraction (by flow cytometry) *Mean* ± *SD* %
*Set 1 Cam*	Camptothecin (6 µM)	34	AO/PI	R/G	8.0 ± 1.0
*Set 1 Ctrl*	No	27	AO/PI	R/G	1.8 ± 0.2
*Set 2 Cam*	Camptothecin (6 µM)	41	Dio/PI	R/(R + G)	8.0 ± 1.0
*Set 2 Ctrl*	No	39	Dio/PI	R/(R + G)	1.8 ± 0.2
*Set 3 Cam*	Camptothecin (6 µM)	31	AO/PI	R/G	10.1 ± 1.2
*Set 3 Ctrl*	No	20	AO/PI	R/G	2.4 ± 0.8
*Set 4 Cam*	Camptothecin (6 µM)	30	Dio/PI	R/(R + G)	10.1 ± 1.2
*Set 4 Ctrl*	No	30	Dio/PI	R/(R + G)	2.4 ± 0.8

AO/PI is Acridine Orange/Propidium iodide, Dio is DioC6/Propidium iodide.

## Data Records

The data record has been deposited at *figshare*^8^. General description of the image set and its general characteristics are summarized in Table 1. Each sample in the image series contains three different images:

1. the optical channel image (file name: “<Set>/<k>0.0.jpg”),
2. the red fluorescence channel image (file name: “<Set>/<k>0.1.jpg”),
3. the green fluorescence channel image (file name: “<Set>/<k>0.2.jpg”).

Were “<k>” a sample number in a series, “<Set>” a folder name for the series according to the Table 2.

**Table 2:** Accordance between series name and folder name.

Series Name	Series Folder
*Set 1 Cam*	Set_1_Cam_AO_PI_19_02_16
*Set 1 Ctrl*	Set_1_Ctrl_AO_PI_19_02_16
*Set 2 Cam*	Set_2_Cam_Dio_PI_19_02_16
*Set 2 Ctrl*	Set_2_Ctrl_Dio_PI_19_02_16
*Set 3 Cam*	Set_3_Cam_AO_PI_19_11_15
*Set 3 Ctrl*	Set_3_Ctrl_AO_PI_19_11_15
*Set 4 Cam*	Set_4_Cam_DIO_PI_19_11_15
*Set 4 Ctrl*	Set_4_Ctrl_DIO_PI_19_11_15

The red channel indicates dead cells in all provided types of staining. The green channel indicates either all cells or viable cells only with *AO/PI* and *Dio* staining, respectively. Thus, all cells can be estimated as sum of the green and the red channels in all cases.

## Technical Validation

On the same plate which has been used for microscopy, cells from a series of 3 wells were treated with trypsin for detachment, the obtained suspension has been stained with DioC6 (0.02 *μg/ml*) and propidium iodide (3 *μg/ml*) and the fraction of non-viable cells has been evaluated with flow cytometry using a BD FACSCanto II flow cytometer. Data analysis was carried out using FACSDiva software.

Due to methodological differences between flow cytometry and microscopy, absolute values of cell population fraction may differ. Therefore, we show the relative increase in the dead cells fraction in samples treated with Cam compared to untreated control, see Table 3. Calculations of the dead cells fraction ratios from fluorescence microscopic images have been performed *in silico* using BioFilmAnalyzer software^9^.

**Table 3:** Types and count of microscopy view fields in the dataset.

Series Name	Dead cells fraction increase, Cam/Ctrl ratio, fold, *Mean* ± *SD*	
	Evaluated by flow cytometry	Evaluated *in silico* based on differential fluorescence microscopy
*Set 1*	4.5 ± 0.9	4.4 ± 2.2
*Set 2*	4.5 ± 0.9	4.0 ± 2.3
*Set 3*	4.7 ± 2.2	4.9 ± 3.1
*Set 4*	4.7 ± 2.2	5.0 ± 2.8

AO/PI is Acridine Orange/Propidium iodide, Dio is DioC6/Propidium iodide

## Usage Notes

Although we provide and use 3-channel color images for optical channel, we recommend converting to grayscale format before analyzing.

We provide a baseline regression example obtained using the U-Net neural network^10^. The segmentation algorithm learning and validation procedures consisted of the following steps.

### Image pre-processing

**Fig. 2:**
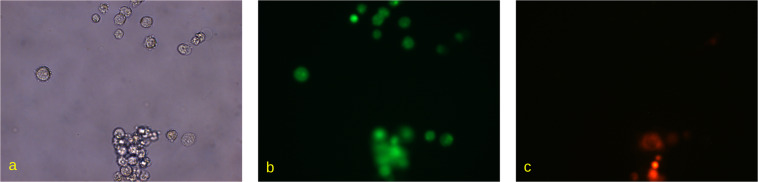
The microscopic images of Caco-2 cells in (**a**) bright-field view, (**b**) green and (**c**) red fluorescent channels. Cells were stained with Acridine Orange and Propidium Iodide and visualised on Carl Zeiss Observer 1.0 microscope with 40× magnification.

**Fig. 3:**
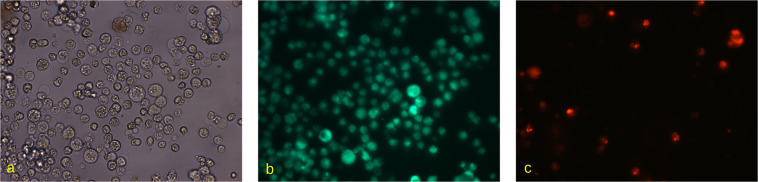
The microscopic images of Caco-2 cells in (**a**) bright-field view, (**b**) green and (**c**) red fluorescent channels. Cells were stained with DioC6 and Propidium Iodide and visualised on Carl Zeiss Observer 1.0 microscope with 40× magnification.

The image set for the experiment contained microscopic images 1388×1040 pixels and the corresponding labels. The image set was first divided into the training set and the validation set at 8:2 ratio. Figures 2, 3 show representative microscopic images with corresponding labels.

For both network training and validation purposes, the green and the red fluorescent channels were used as reference images, representing physical markup of the viable and non-viable cells, respectively. Next the green and the red channels for each field of view have been merged into single color image, and the blue channel has been filled with zeros. Due to the large size of the image, the images have been resized to size 512×512 due to the limitations of the neural network. For the same reasons, the images have been standardized and normalized, with the aim of enhancing the network convergence rate and also improving the segmentation accuracy.

In addition, to make full use of the finite image set, we perform the following training-time transformation: a random horizontal flip with a given probability and a random 90° rotation image set have also been expanded with the addition of Gaussian noise and random smoothing. Thus, the final generalization ability of the model is improved. Additionally, for the input images themselves, the data enhancement process including the addition of Gaussian noise had also been implemented.

### Regression model

Next, U-Net was trained using AdamW optimizer^11^ with learning rate 10^−4^ and weight decay 10^−5^. The neural network used has three input channels (optical RGB) and two output channels (corresponding to the virtual “red” and “green” staining, denoted as R and G, respectively). As a loss function and target quality metric, MSELoss was utilized, which measures the mean squared error (squared L2 norm) between each element in the input *x* and the target *y*.

1 $$\ell (x,y)=mean\left({\left\{{l}_{1},\ldots ,{l}_{N}\right\}}^{{\rm{T}}}\right),\quad {l}_{n}={\left({x}_{n}-{y}_{n}\right)}^{2}$$

where *x* and *y* are arbitrary shapes tensors with a total of *n* elements each, *N* is the batch size.

**Fig. 4:**
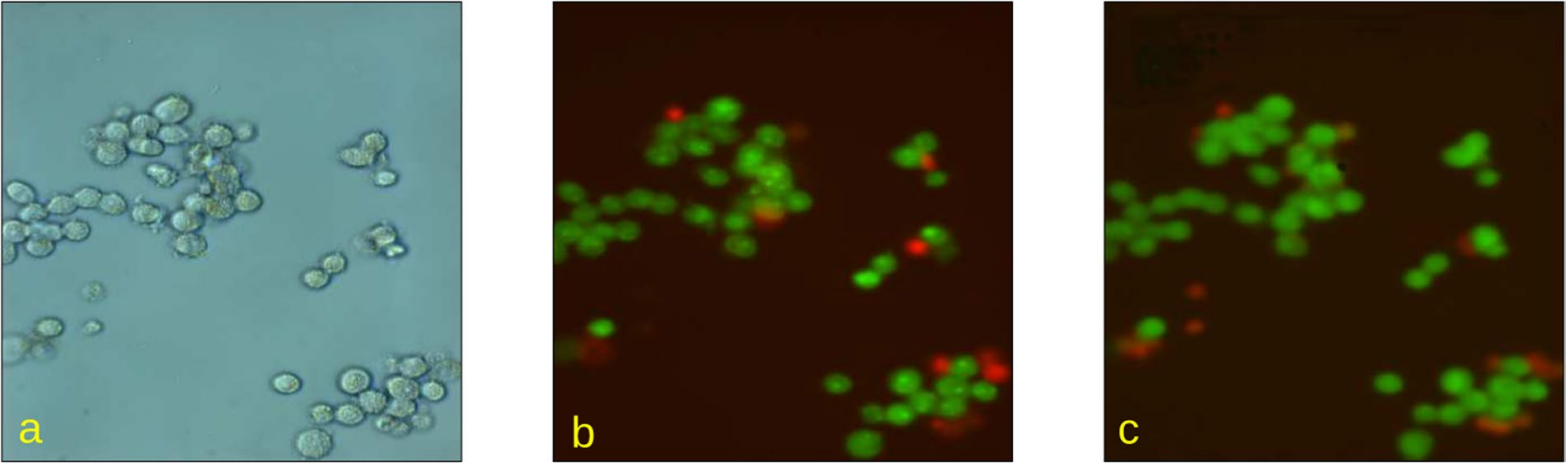
The microscopic images of Caco-2 cells in (**a**) bright-field view and (**b**) overlapped green and red fluorescent channels, as well as (**c**) the corresponding “virtual staining” image obtained using prediction based on the U-Net based regression neural network model as indicated above.

The model was trained for totally 1000 epochs with unit batch size. A single representative example of the prediction results placed side-by-side with the respective fluorescent staining image is shown in Fig. 4.

### Quality estimation

**Fig. 5:**
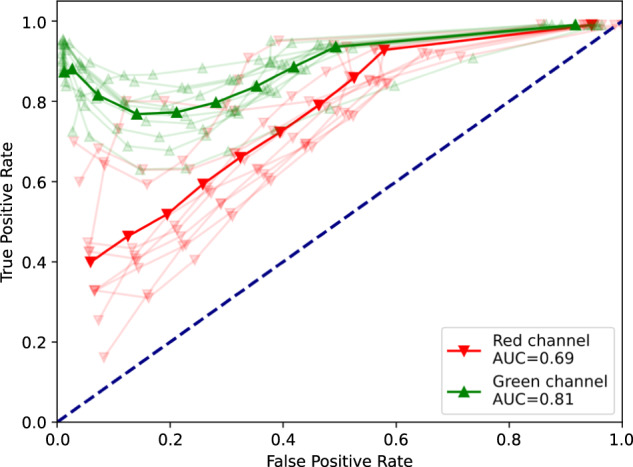
Transmitter-Receiver Operating Characteristic (TROC) curves characterizing the agreement between the physical and virtual staining channels obtained by multi-threshold segmentation with simultaneous adjustment of thresholds to fit the same quantiles of the physical and the virtual channel intensity distributions, respectively.

To validate how accurately the virtual staining images reproduce the results obtained by physical staining, we further compared them using fixed-threshold segmentation scenarios for a series of thresholds corresponding to fixed image quantiles. The segmentation threshold has been applied simultaneously to both physical and virtual staining images, areas with image intensities exceeding the threshold related to the same quantile in both channels the have been selected, and the overlap between the selected areas has been calculated to quantify the match between the segmentation procedures based on the physical and on the virtual channels, respectively. The segmentation accuracy for a series of thresholds for ten different images, as well as their overall average, are shown in Fig. 5 in the form of the Transmitter-Receiver Operating Characteristic (TROC) curve, designed similarly to the conventional Receiver Operating Characteristic (ROC) curve, except for the threshold adjustment that is performed not only at the receiver, but also simultaneously at the transmitter side. Areas under the curve (AUC) for the average TROC curves are 0.81 for viable cells and 0.69 for dead cells, respectively. Remarkably, True Positive Rate (TPR) exceeds 0.9 not only for high, but also for low threshold values (corresponding to the quantiles at both tails of the distribution), indicating similarly efficient segmentation of areas covered with cells, as well as cell-free areas.

## Data Availability

The source code of the baseline, as well as a direct link to the VirtualStaining Dataset, is available in the GitLab repository^12^. The installation of Python and Jupyter using the virtual environment is recommended, with the necessary technical instruction supplied in the “ReadMe.md” inside the repository.
